# Re-testing reported significant SNPs related to suicide in a historical high -risk isolated population from north east India

**DOI:** 10.1186/s41065-020-00144-y

**Published:** 2020-07-17

**Authors:** Gaurav Gupta, Ravi Deval, Anshuman Mishra, Shashank Upadhyay, Piyoosh Kumar Singh, V. R. Rao

**Affiliations:** 1 Department of Biotechnology, Invertis University, Bareilly (U.P), India; 2 Department of Genetics, Osmania University, Hyderabad, 500007 India; 3 VBRI Innovation Centre, New Delhi, India; 4 Institute of Advanced Materials (IAAM), 59053 Ulrika, Sweden; 5 Department of Anthropology, Delhi University, Delhi, India; 6 Department of Psychiatry, All India Institute of Medical Sciences, New Delhi, India; 7 Genome Foundation, Hyderabad, India

**Keywords:** Suicide, GWAS, SNPs, Replication

## Abstract

**Background:**

Genetic diathesis of suicide is supported by family and twin studies. Few candidate gene pathways are known, but does not explain fully the complexity of suicide genetic risk. Recent investigations opting for Genome-Wide Association Studies (GWAS) resulted in finding additional targets, but replication remained a challenge. In this respect small isolated population approach in several complex disease phenotypes is found encouraging. The present study is an attempt to re-test some of the reported significant SNPs for suicide among a small historical high- risk isolated population from Northeast India.

**Methods:**

Two hundred ten cases (inclusive of depressed, suicide attempter and depressed + suicide attempter) and 249 controls were considered in the present study which were evaluated for the psychiatric parameters. Sixteen reported significant SNPs for suicide behaviour were re-tested using association approach under various genetic models. Networking by GeneMANIA tool was used for function prediction of the associated genes.

**Results:**

Seven SNPs (of 6 genes) remained significant in different genetic models. On networking genes with significant SNPs *IL7, RHEB, CTNN3, KCNIP4*, *ARFGEF3* are found in interaction with already known candidate gene pathways while SNP rs1109089 *(RHEB*) gained further support from earlier expression studies. *NUGGC* gene is in complete isolation.

**Conclusions:**

Small population approach in replicating significant SNPs is useful in complex phenotypes like suicide. This study explored the region-specific demographics of India by identifying vulnerable population for suicide via genetic association analysis in bringing into academic and administrative forum, the importance of suicide as a disease and its biological basis.

## Introduction

Suicide represents a wide range of risk factors encompassing throughout the life period of the subjects. Recently, suicide is considered as a major public health issue and is the second leading cause of death globally among the age groups of 15 to 29 years [1]. Almost 800,000 suicides are completed every year and in this 79% are documented from the low and middle-income countries alone [2]. Suicide death rate in India from 1990 to 2016 was estimated at 17.9 per 100,000 persons, which equates to around 230,000 suicides annually [3]. The complex etiology of suicide involves interaction of genetics with other psychiatric, neurological and environmental conditions. Twin studies demonstrated that suicide has a genetic component and familial basis is evident and is now proven that greater risk of a suicide attempt is involved in the offspring of a candidate with a positive history of completed suicide [4]. Besides, the increased rate of suicide attempt is found in the first-degree relatives of suicide probands [5]. However, the daunting task of complexity lies in the partial genetic contribution of other associated psychiatric disorders such as mood, alcohol/ substance use, schizophrenia etc. along with independent heritable factors for impulsivity, aggression [6]. As of now, few pathways and candidate genes in the central nervous system, serotonergic hypofunction and impaired negative feedback of the hypothalamic-pituitary-adrenal axis are frequently observed in both attempters and those who die by suicide [7] . The contribution of these candidate gene association studies underscores the complexity of suicide genetic risk, leading to several recent investigations opting for hypothesis-free methods like Genome-wide association studies (GWAS) of common variants [8–13]. As a result, many known and novel variants have been found [14, 15]. However, replication of the suggestive significant GWAS SNPs in other cohorts remains a challenge [8, 13]. Factors such as sample size [16], phenotype cohort [17], correlation with family history [18], subject follow-ups, lifestyle and cultural differences from population to the population sampled [19], all these together play a vital role in failing to replicate the significance of a GWAS SNP in the pathogenesis of suicide [8, 18]. On the other hand, small isolated populations are known to be useful in replication, as they yield better results even if the sample size is relatively small as compared to large sample size requirements in heterogeneous large populations. Besides, similar co-factors (environment, lifestyle, geography, ethnicity) between case/control holds a benefit along with the advantage of founder effect and reduced genetic heterogeneity in small isolated populations, which is a challenge in general population screening even if sample numbers are large [20]. It is also observed that due to founder effect the frequency of phenotypic traits of a complex disease is likely to be high in small populations [21].

The Idu-Mishmi is an isolated small endogamous Tibeto-Burman speaking tribe inhabiting Lower and Upper Dibang Valleys in Arunachal Pradesh, India and numbered around 15,000 souls in 2001 census [22]. Mene [23–25] reported that, between 1971 and 2010, 218 cases of suicide occurred in this tribe in the 10–29 age group with an estimated suicide rate of 58 per 100,000 individuals surpassing the national average. In a systematic sampling and phenotypic annotation, our studies on Idu- Mishmi reported a high rate of attempted suicide (14.2% compared to the general urban population frequency of 0.4–4.2%) and significant association of depression and endo-phenotypes (impulsivity and aggression) [26–28]. The key objective of the present study is whether suggestive significant SNPs reported in GWAS studies conducted in 10 years period (2004 to 2015) and published in a comprehensive review [8], targeting suicide behavior can be replicated in the historical isolated small Idu-Mishmi population with a high rate of suicide attempt.

## Methods

The study subjects recruited were based on their psychiatric trait assessment of suicide attempt, depression and family history. The controls were negative for both psychiatric traits screened and family history. Idu-Mishmi is a close-knit community distributed over a few settlements and suicide occurrences are a shared memory of 3 to 4 generations accordingly, families can be identified. To control the relatedness in a small endogamous population sampling was done on critical screening where primary and secondary degree relatives were not included in the study. The psychiatric assessment of suicide behavior was done based on the Columbia Suicide Severity Rating Scale (C-SSRS) and PHQ-9 used for depression assessment. The reliability and validity of these scales used were described in our study [26, 27]. All the studied individuals with a suicide attempt, depression or both with or without positive family history have been considered as cases (*N* = 210) and 249 healthy controls were recruited without any of the given traits and negative family history. The sample numbers for genotyping vary between SNPs due to degradation in storage and transportation of blood samples from remote areas.

The suggestive SNPs with significant threshold at *p* < 0.001 [8] were selected and included in the present study. Genotyping in the study population was done with ARMS PCR Technique and SNP location traced based on coordinate position (GRCh38.p7) followed by sequence retrieval from ensemble genome browser [https://asia.ensembl.org/index.html]. Formatting of the retrieved sequences was done using ApE (v2.0.55). [http://jorgensen.biology.utah.edu/wayned/ape/]. Forward and reverse (normal and mutant) primers were designed using Primer3Plus [http://www.bioinformatics.nl/cgi-bin/primer3plus/primer3plus.cgi]. To increase the specificity, weak secondary mismatches were incorporated at the penultimate position manually for a strong primary mismatch and a strong secondary mismatch was introduced for a weak primary mismatch for prevention of any false positives as two mismatches PCR will not continue and in case of one mismatch, the PCR will be initiated [29]. All the 16 primer sequences used are shown in Table S1. Each primer was standardized for specific Tm and specificity using gradient PCR then validation PCR was carried out on 20 samples to check for specificity and Tm. To ensure the selectivity and specificity re-validation of same samples were done twice with similar conditions to ensure similar genotypes in each run. In-silico PCR was also carried out for all the primers and expected band sizes were carefully observed for the exclusion of any false positives. Extreme care was taken in designing and validation, after a complete testing only validated primers were used for genotyping by ARMS technique for all the cases and controls. Genotyping was performed using agarose gel electrophoresis on a 2% gel stained with ethidium bromide and visualized in T-Genius, Syngene gel documentation system.

Allele frequencies were calculated by gene counting method and HW equilibrium test by on line portal (https://wpcalc.com/en/equilibrium-hardy-weinberg/). Differences in age and sex among cases and controls were presented as percentages with Z test and p- values. Inheritance models (dominant, recessive and additive) were employed for the categorization and interpretation of the data. Age, sex-adjusted bivariate analyses were performed using the SPSS package (v16.0, SPSS Chicago). Multiple testing by Bonferroni correction was performed on the significant SNPs to remove false positives, the critical value was set to 0.05 and the number of tests to 25 and the corrected critical value was kept 0.002. Network analyses were done by GeneMANIA (https://genemania.org/), a user- friendly flexible web site that uses a wealth of genomics and proteomics data and finds functionally similar genes with a gene list query [30]. The default parameters of the network with a percentage contribution of each are as given in Table S4. This study was approved by the Institutional Ethical Board of the Department of Anthropology, Delhi University and all subjects participated in the study gave their written informed consent.

## Results

Table 1 shows age, sex distribution in cases and controls of the study. Overall females are at slightly higher risk (*p* = 0.08186), however between age groups > 19 years show higher risk in both sexes, with significant difference in males (*p* = 0.0004). Information on each of the selected SNPs, their genomic context within the respective genes, associated psychiatry traits reported and minor allele frequencies (MAF) of the present population in comparison to other continental populations available from European, South East Asian and South Asian populations are given in Table S2. The variation of MAF frequency in controls is within the range reported for East Asian and South Asian populations. Genotypes and allele frequencies of the 16 selected SNPs with HW test of significance along with a percentage of the difference between observed and expected heterozygotes are shown in Table S3. Ten of 16 SNPs are with significant HW deviation among controls with low observed heterozygote frequencies. Bivariate analyses with age-sex adjusted ODDs ratios in dominant, recessive and additive genetic models were computed and 7 SNPs (of 6 genes) remained significant with Bonferroni corrected *p*-values (Table 2). Significant SNPs of genes *RHEB, NUGCC* are at risk in the dominant model, IL7 in the recessive model, *ARFGEE3, KCNIP4* in the additive model and *CTNN3* in dominant and recessive models. However, simultaneous protection in alternative models is also seen. All are at a higher threshold of significance (*p* < 2.50E^− 08^), whereas ARFGEE3 gene SNP at *p* < 0.035. Figure S1 shows the networking of 6 genes with significant found SNPs in our population along with candidate genes of serotonergic system (*TPH1, TPH2, HTR1A, HTR1B, HTR2A, SLC6A4*), dopaminergic and adrenergic system (*DRD2, AKT1, AKTIP, ADRA2*), catabolism of monoamines system (*COMT, MAOA*), *HPA* axis pathway (*CRHR1, CRHR2, FKBP5, CRHBP, NR3C1, AVPR1B*) and neurotrophic processors (*BDNF, TRKB, CCKBR, NGF, NTRKR2, HOMER1, NPTX2*). Five out of 6 genes were observationally found interacting/closer with specific candidate gene pathways, i.e. *KCNIP4* (Neurotrophic processors), *RHEB (HPA* axis), IL7*& CTNNA3* (Serotonergic), *ARFGEF3* (Dopaminergic and Adrenergic). However, *NUGGC* gene is having no interaction with any of the genes in the network and is lying in distant isolation.

**Table 1:** Age-sex distribution among cases and controls along with Z-test values

Categories	Male		Female	
	< 19.0 years	> 19.1 years	< 19.0 years	> 19.1 years
Cases	^a^33 (29.73%)	^b^68 (52.31%)	^c^49 (46.23%)	^d^60 (53.57%)
Controls	78 (70.27%)	62 (47.69%)	57 (53.77%)	52 (46.43%)
Total	111	130	106	112

Z Test (*p* values): a*b = −3.5411(0.0004); c*d = −1.0833(0.28041); a*c = −2.5058(0.01208); b*d = − 0.1958(0.84148)

**Table 2:** Association analysis of SNPs in different genetic models (age and sex adjusted) between cases and controls with Bonferroni corrected *p* -values

Case/Control (Age-Sex adjusted)			
rsID (Gene)	Dominant Model	Recessive Model	Additive Model
	Odds Ratio	Odds Ratio	Odds Ratio
	(Lower-Upper)	(Lower-Upper)	(Lower-Upper)
	CI- 95%	CI- 95%	CI- 95%
rs358592 (Risk = C) (*KCNIP4*)	0.268	0.343	5.174
	(0.165–0.434) (*p <* 2.50E^− 08^)	(0.143–0.823) (*p =* 0.425)	(2.094–12.788) (*p* < 2.50E^− 08^)
			Heterozygous
rs1109089 (Risk = T) (*RHEB*)	3.477	2.047	0.268
	(2.137–5.656) (*p* < 2.50E^− 08^)	(1.101–3.805) (*p =* 0.575)	(0.136–0.531) (*p* < 2.50E^− 08^)
			Heterozygous
rs10448044 (Risk = C) (*IL7*)	2.091	3.314	0.306
	(1.27–3.442) (*p =* 0.1)	(1.993–5.509) (*p* < 2.50E^−08^)	(0.175–0.535) (*p* < 2.50E^− 08^)
			Heterozygous
–	–	–	0.293
			(0.15–0.575) (*p* < 2.50E^−08^)
			Homozygous
rs4732812 (Risk = T) (*NUGCC*)	8.122	1.457	0.201
	(4.002–16.483) (*p* < 2.50E^−08^)	(0.639–3.32) (*p =* 1)	(0.076–0.531) (*p =* 0.025)
			Heterozygous
rs10448042 (Risk = G) (*IL7*)	1.205	5.097	0.273
	(0.666–2.179) (*p =* 1)	(2.53–10.27) (*p* < 2.50E^−08^)	(0.13–0.572) (*p =* 0.025)
			Heterozygous
–	–	–	0.097
			(0.038–0.251) (*p* < 2.50E^−08^)
			Homozygous
rs10997044 (Risk = A) (*CTNNA3*)	12.363	36.591	0.026
	(5.236–29.192) (*p* < 2.50E^−08^)	(9.92–134.974) (*p* < 2.50E^− 08^)	(0.007–0.096) (*p* < 2.50E^− 08^)
			Heterozygous
–	–	–	0.052
			(0.009–0.301) (*p =* 0.025)
			Homozygous
rs203136 (Risk = G) (*ARFGEF3*)	0.331	0.944	2.416
	(0.152–0.719) (*p =* 0.005)	(0.498–1.787) (*p =* 0.859)	(1.068–5.462) (*p =* 0.034)
			Heterozygous

## Discussion

The present study is born out of the fact that significant SNPs in several GWAS studies conducted in various populations, failed to replicate in other populations/cohorts because of various factors like large sample size requirement, phenotypic heterogeneity and lifestyle cultural differences etc. This remained a major challenge in understanding the pathogenesis of suicide, in-spite of the fact that these SNPs may be important and may provide an opportunity for discovery of novel bio-pathological pathways or strengthening known pathways in biomarker discovery for suicide risk and therapeutic intervention. Founder effect events in historically isolated small populations are useful in finding genes not only in Mendelian disorders but also in polygenes of several complex disease phenotypes with the advantage of narrowing down on the sub-phenotype heterogeneity [31–34]. To address this issue, we designed the present study in a historical small isolated endogamous Idu Mishmi population having the highest rate of suicide attempt (14.2%) compared to general urban population (0.4–4.2%) with depression as a significant covariate described in our earlier studies [26, 27]. Genetic variants identified in a small population are not restricted only by founder effects, but they can also be mapped in larger populations that help in identification of new or strengthening known pathways underlying the effect of these SNPs in other complex diseases [20]. The Human genome has been explored to have around 10 million variants/SNPs varying individually [35], therefore it becomes a strong point to consider GWAS SNPs in the pathobiology of suicide. Some of the reported suggestive significant SNPs for suicide were chosen for re-testing in our small high-risk isolated population association study on the subjects recruited after careful evaluation of psychiatric traits, suicide attempt and depression, and we found several SNPs were highly significant.

In pathway analysis by GeneMANIA (https://genemania.org/) the suggestive significant SNP genes are associated with specific biological networks (pathway, co-expression, shared protein domains, physical interaction, co-localization, genetic interaction) of the candidate genes, observationally. *RHEB, CTNNA3, KCNIP4, IL7, ARFGEF3* were interacting with the candidate genes via connecting genes/pathways. *RHEB* gene product is a GTP-binding protein called *RAS* homolog enriched in the brain. The main function of this gene is involved in *mTOR* pathway and regulation of the cell cycle. The SNP of this gene (rs1109089) which has been re-tested and found significant in the present study, has already been implicated in suicide [36]. *mTOR* pathway is well known for its antidepressant drug response [37–39]. Emerging quick and effective antidepressants are now available based on *RHEB* mediated mTOR pathway which is showing an effect on treatment-resistant subjects [40, 41]. *CTNNA3* gene has roles implicated in the formation of stretch-resistant cell-cell adhesion complexes and is reported for causing mental ailments such as schizophrenia [42], besides the gene is reported to be expressed in the cerebellum [43]. *KCNIP4* gene is found significant for various drug targets being a *Kv* channel-interacting protein 4 family [44]. *IL7* gene in the immune system plays a role in psychiatric disorders is a fact and a lot of research claims this point, along with other cell types, it is also produced in neurons and it has close interconnections with serotonergic pathways via *IL9* as shown in figure S1. A study reported high expression levels of *IL7* in affected males and low levels in affected females [45]. *ARFGEF3* gene showed connection via a connecting gene to the candidate gene *DRD2* of a dopaminergic and adrenergic pathway. The genes with significant variants found suggestive of their role in the pathogenicity of suicide attempt and depression in the present study. However, more insights are required to explore functional validation of the role of these Genes/SNPs in causing the phenotypes considered.

The most significant observation of the study is *NUGGC* gene (Nuclear *GTPase* SLIP- GC) with significant risk allele in our bivariate analysis, is an outlier in the network (Figure S1). *NUGGC* inhibits function of the activation-induced cytidine deaminase *AICDA* [46] and helps in maintenance of genome stability by reduction of somatic hyper-mutations in B-Cells [47]. The functional validation of this gene may lead to discovery of entirely new bio-pathway in suicide research.

## Conclusion and future prospects

Worldwide suicide is considered as the leading cause of death. This study explores the region-specific demographic assessment of the suicidal genetic risk. Re-testing of reported significant SNPs in our study population and functional correlation with known candidate gene bio-pathways contributed to the advantage of historically isolated populations in deciphering genetics of complex phenotypes like suicide. Having such a small population with high prevalence of the targeted phenotype is an added advantage. Besides, the finding of involvement of a gene related to genome stability may be important in finding entirely novel bio-pathways in future suicide research. However, the findings of this study need to be considered as exploratory and further functional validations are definitely in need.

Limitations: Subject selection is a challenge in a small population genetics association study. Significant HW deviation is a concern, low observed heterozygote frequencies are as expected in local small endogamous population. However, 3 genes with suggestive significant SNPs re-tested in our study were in HW equilibrium for controls. In studies aiming re-testing or replication of previous studies, one of the serious limitations is different methods of phenotypic annotation used, which also applies to the present study.

## Supplementary information

**Additional file 1: Table S1.** Primer sequences used for genotyping along with melting temperature and amplicon base pairs. **Table S2.** List of suggestive significant SNPs chosen from a review of 10 year study for re-testing in the present study. **Table S3.** Distribution of genotypes, allele frequencies and HW test of significance among cases and controls of the 16 selected SNPs in the present study. **Table S4.** Network pathway percentage contribution of different default parameters of the figure S1. **Figure S1.** Pathway network analysis of 6 significant genes of the present study along with candidate genes of different known pathways of suicide behaviour. Default settings used for creation of network are as described in Table S4.

## Data Availability

All the analysis data will be made available upon request.

## Abbreviations

GWAS: Genome Wide Association Study
SNP: Single Nucleotide Polymorphism
C-SSRS: Columbia Suicide Severity Rating Scale
PHQ-9: Patient Health Quesstionaire-9
ARMS: Amplification Refractory Mutation System
PCR: Polymerase Chain Reaction
Tm: Melting Temperature
HW: Hardy–Weinberg
MAF: Minor Allele Frequency
