# Supplementary material for: Re-testing reported significant SNPs related to suicide in a historical high -risk isolated population from north east India
Source: Hereditas. 2020 Jul 17;157:31. doi: 10.1186/s41065-020-00144-y (PMC7368720; doi:10.1186/s41065-020-00144-y)
Supplement: Supplementary file 1 — Additional file 1: Table S1. Primer sequences used for genotyping along with melting temperature and amplicon base pairs. Table S2. List of suggestive significant SNPs chosen from a review of 10 year study for re-testing in the present study. Table S3. Distribution of genotypes, allele frequencies and HW test of significance among cases and controls of the 16 selected SNPs in the present study. Table S4. Network pathway percentage contribution of different default parameters of the figure S1. Figure S1. Pathway network analysis of 6 significant genes of the present study along with candidate genes of different known pathways of suicide behaviour. Default settings used for creation of network are as described in Table S4. [file 41065_2020_144_MOESM1_ESM.docx]

**Supplementary Data:**

**Table S1: Primer sequences used for genotyping along with melting temperature and amplicon base pairs.**

| **rsID** | **Forward primer 5’-3’** | **Reverse primer 5’-3’ (Normal)** | **Reverse primer 5’-3’ (Mutant)** | **T_m_ in ^0^C** | **Amplicon (bp)** |
| --- | --- | --- | --- | --- | --- |
| **rs358592** | GCATTTCGAGGACTGTTCCAGAG | GCTTAGAGAAGATGTGACAGAAGTGAA**T**GC | GCTTAGAGAAGATGTGACAGAAGTGAA**T**GT | 69.5 | 351 |
| **rs1109089** | GCTCCTTGCCAGGTGCTTCACTTC | CGGTTGCATTACAGAACCACGGTTTCC**G**CC | CGGTTGCATTACAGAACCACGGTTTCC**G**CT | 70.5 | 355 |
| **rs4918918** | GACTCCTTCCTGCAAAGCCACAGTT | CAACCGCCTTCTAGACATCCACCTGAA**G**GT | CAACCGCCTTCTAGACATCCACCTGAAGGC | 69.5 | 535 |
| **rs10448044** | ATGGCAGTCCATATCTGCCACTTG | CCAATAGTGGCAGCAGTTCCTGTGGCC**T**CA | CCAATAGTGGCAGCAGTTCCTGTGGCC**T**CG | 69.5 | 352 |
| **rs4732812** | CAAGGGTATCAACGTCTTTGCCA | ACGGAGGAGATAATAAAAGCTCTTTAA**C**AC | ACGGAGGAGATAATAAAAGCTCTTTAA**C**AT | 66.5 | 310 |
| **rs10448042** | GTGCTCTTCACCAACAGCAGTC | CCTCAAGCAGGCCCCAGTGTCTGCTGT**G**TT | CCTCAAGCAGGCCCCAGTGTCTGCTGT**G**TC | 70.5 | 601 |
| **rs10997044** | ACTTCCAAATGCCTTGTGTCTCCT | TTCAAAAGAGATGTTTCTTATTGTCT**T**CG | TTCAAAAGAGATGTTTCTTATTGTCT**T**CA | 70.5 | 648 |
| **rs203136** | TTCTGAGAAATGCATTGTTAGGCA | TTTCACTTGATCATCGAAACAG**A**TA | TTTCACTTGATCATCGAAACAG**A**TC | 70.5 | 352 |
| **rs1360550** | CCAGACTCCAGGTGCAGGAGAA | CTTCCAGGCTGTTCCAGCAATGCTTAG**A**CC | CTTCCAGGCTGTTCCAGCAATGCTTAG**A**CT | 65.5 | 240 |
| **rs1037448** | GTGCTAGGATTACAGGCGTGAGC | GCATTAGCCATGGGGCACTGATGCCTA**T**GA | GCATTAGCCATGGGGCACTGATGCCTA**T**GG | 65.5 | 362 |
| **rs2576377** | GTCCTGTGCACAGGAGGTGGATTA | TCATAATGCTCAAGTTGGGGTGCCAAC**G**GC | TCATAATGCTCAAGTTGGGGTGCCAAC**G**GT | 69.5 | 499 |
| **rs4751955** | TGACCCATAAGAGGAACCTGTTCAT | GGCTCACAAACTTACCGTGTGCATTC**A**AT | GGCTCACAAACTTACCGTGTGCATTC**A**AC | 65.5 | 631 |
| **rs11143230** | GTCCATTGGAAAGCTATCTCTAAC | CTAGATTTCCAAGCCTTGACTCTTAA**G**AT | CTAGATTTCCAAGCCTTGACTCTTAA**G**AG | 65.5 | 355 |
| **rs3851150** | TGTGCTTCAGCTACTTCTCCAG | AAATATGTTTACATTTACCTTCAT**G**TT | AAATATGTTTACATTTACCTTCAT**G**TG | 59.5 | 390 |
| **rs10854398** | AGCTGGAGGTAGGAGGTGAAGACA | AGTTGGTTTTGCCAGGATCCCACACCA**G**CA | AGTTGGTTTTGCCAGGATCCCACACCA**G**CG | 59.5 | 372 |
| **rs11628713** | CGGAAGCGGAGCGTTACTTGCCGG | AGGACATGCTTGCTGGGTTTGTAGC**T**AG | AGGACATGCTTGCTGGGTTTGTAGC**T**AA | 65.5 | 555 |

* Incorporated Second mismatches are highlighted in bold in the reverse normal and reverse mutant primer sequences.

**Table S2: List of suggestive significant SNPs chosen from a review of 10 year study for re-testing in the present study**

| **rsID** | **Risk Allele** | **MAF** | | | | ***p*- value** | **n** | **Gene** | **Intronic/**  **Exonic** | **ethnicity** | **Psychiatry Trait** | **Significance reported by** |
| --- | --- | --- | --- | --- | --- | --- | --- | --- | --- | --- | --- | --- |
|  |  | **1000**  **Genomes** | **MAF (EUROPEAN)** | **MAF**  **(ASIAN)** | **Present**  **Study**  **(Controls)** |  |  |  |  |  |  |  |
| **rs358592** | C | 0.309 | Estonian= 0.270  Northern Sweden= 0.353 | South Asian  SAG=0.605  SGDP=0.686  SSIP=0.722  1000G SAS=0.632  WGP=0.692  PGA=0.850  South East Asian  Vietnamese= 0.380 | 0.412 | 2.50X10^–6^ | 706 | *KCNIP4* | Intron variant,  genic upstream transcript variant | European | unipolar depression and suicide ideation | Perroud et al. (2012) |
| **rs1109089** | T | 0.395 | Estonian= 0.490  Northern Sweden= 0.453 | South Asian  SAG=0.579  SGDP=0.426  SSIP=0.638  1000G SAS=0.622  WGP=0.795  PGA=0.600  South East Asian  Vietnamese= 0.212 | 0.275 | 1.67X10^-5^ | 898 | *RHEB* | Intron variant | European | Major Depression, Bipolar Disorder and Suicide Ideation | Menke et al. (2012) |
| **rs4918918** | T | 0.362 | Estonian= 0.384  Northern Sweden= 0.411 | South Asian  SAG=0.730  SGDP=0.577  SSIP=0.736  1000G SAS=0.713  WGP=0.800  PGA=0.800  South East Asian  Vietnamese= 0.333 | 0.574 | 3.28X10^-6^ | 2805 | *SORBS1* | Intron variant | US, Caucasian Mixed | Depression, Bipolar Disorder and Suicide attempt | Perlis et al. (2010) |
| **rs10448044** | C | 0.373 | Estonian= 0.229  Northern Sweden= 0.228 | South Asian  SAG=0.315  SGDP=0.392  SSIP=0.250  1000G SAS=0.323  WGP=0.454  PGA=0.400  South East Asian  Vietnamese= 0.210 | 0.420 | 2.81X10^-6^ | 1123 | *IL7* | Intron variant | Canadian, European | Bipolar Disorder leading to Suicide attempt | Zai et al. (2015) |
| **rs4732812** | T | 0.239 | Estonian= 0.261  Northern Sweden= 0.306 | South Asian  SAG=0.273  SGDP=0.220  SSIP=0.208  1000G SAS=0.241  WGP=0.350  PGA=0.500  South East Asian  Vietnamese0.165 | 0.252 | 3.35X10^–6^ | 706 | *NUGGC* | Intron variant,  genic upstream transcript variant | European | unipolar depression and suicide ideation | Perroud et al. (2012) |
| **rs10448042** | G | 0.373 | Estonian= 0.230  Northern Sweden= 0.228 | South Asian  SAG=0.315  SGDP=0.327  SSIP=0.250  1000G SAS=0.322  WGP=0.454  PGA=0.400  South East Asian  Vietnamese= 0.202 | 0.229 | 3.65X10^-6^ | 1123 | *IL7* | Intron variant | Canadian, European | Bipolar Disorder leading to Suicide attempt | Zai et al. (2015) |
| **rs10997044** | A | 0.216 | Estonian= 0.194  Northern Sweden= 0.185 | South Asian  SAG=0.184  SGDP=0.188  SSIP=.319  1000G SAS=0.231  WGP=0.562  PGA=0.500  South East Asian  Vietnamese= 0.078 | 0.054 | 0.001 | 898 | *CTNNA3* | Intron variant ,genic downstream transcript variant | European | Major Depression, Bipolar Disorder and Suicide Ideation | Menke et al. (2012) |
| **rs203136** | G | 0.425 | Estonian= 0.325  Northern Sweden=0.235 | South Asian  SAG=0.539  SGDP=0.523  SSIP=0.722  1000G SAS=0.624  WGP=0.560  PGA=0.700  South East Asian  Vietnamese= N.A | 0.676 | 1.91X10^-7^ | 2154 | *ARFGEF3* | Intron variant | UK | depression and suicide attempt | Schosser et al. (2011) |
| **rs1360550** | A | 0.380 | Estonian= 0.256  Northern Sweden= 0.403 | South Asian  SAG=0.511  SGDP=0.394  SSIP=0.444  1000G SAS=0.460  WGP=0.625  PGA=0.200  South East Asian  Vietnamese= 0.328 | 0.349 | 8.95X10^-6^ | 2805 | *PRKCE* | Intron variant | US, Caucasian Mixed | Depression, Bipolar Disorder and Suicide attempt | Perlis et al. (2010) |
| **rs1037448** | T | 0.262 | Estonian= 0.100  Northern Sweden= 0.08 | South Asian  SAG=0.877  SGDP=0.820  SISP=0.805  1000GSAS=0.818  WGP=0.850  PGA=0.050  South East Asian  Vietnamese= 0.018 | 0.069 | 1.48X10^-5^ | 898 | *TMEM138* | Intron variant | European | Major Depression, Bipolar Disorder and Suicide Ideation | Menke et al. (2012) |
| **rs2576377** | C | 0.091 | Estonian= 0.171  Northern Sweden=0.165 | South Asian  SAG=0.946  SGDP=0.930  SSIP=0.930  1000GSAS=0.945  WGP=0.946  AG=1.0  South East Asian  Vietnamese= N.A | 0.048 | 2.55X10^-8^ | 2805 | *ABI3BP* | Intron variant,  Genic upstream transcript variant | US, Caucasian Mixed | Depression, Bipolar Disorder and Suicide attempt | Perlis et al. (2010) |
| **rs4751955** | A | 0.475 (A) | Estonian= 0.404  Northern Sweden= 0.496 | South Asian  SAG=0.556  SGDP=0.543  SSIP=0.486  1000G SAS=0.532  WGP=0.695  PGA=0.450  South East Asian  Vietnamese= N.A | 0.243(A) | 7.576X10^-7^ | 2154 | *GFRA1* | Intron variant | UK | depression and suicide attempt | Schosser et al. (2011) |
| **rs11143230** | C | 0.234 (C) | Estonian= 0.294  Northern Sweden= 0.260 | South Asian  SAG=0.264  SGDP=0.238  SSIP=0.208  1000G SAS=0.259  WGP=0.500  PGA=0.350  South East Asian  Vietnamese= N.A | 0.172(C) | 1.54X10^–6^ | 706 | *GDA* |  | European | unipolar depression and suicide ideation | Perroud et al. (2012) |
| **rs3851150** | C | 0.187 (C) | Estonian= 0.195  Northern Sweden= 0.261 | SAG=0.136  SGDP=0.168  SSIP=0.097  1000G SAS=0.117  WGP=0.500  PGA= N.A  South East Asian  Vietnamese= N.A | 0.063(C) | 4.60X10^-6^ | 1123 | *-* |  | Toronto, Canada, UK | Bipolar Disorder leading to Suicide attempt | Zai et al. (2015) |
| **rs10854398** | C | 0.486(C) | Estonian= N.A  Northern Sweden= 0.481 | SAG=0.505  SGDP= N.A  SSIP=0.513  1000G SAS=0.531  WGP=0.625  PGA= 0.600  South East Asian  Vietnamese= 0.070 | 0.405(C) | 6.06X10^-6^ | 2805 | *B3GALT5* | Intron variant,  Genic upstream transcript variant | US, Caucasian Mixed | Depression, Bipolar Disorder and Suicide attempt | Perlis et al. (2010) |
| **rs11628713** | T | 0.135 (T) | Estonian= 0.170  Northern Sweden= 0.180 | SAG=0.184  SGDP=0.150  SSIP=0.125  1000G SAS=0.165  WGP=0.382  PGA=0.350  South East Asian  Vietnamese= 0.070 | 0.365(T) | 6.2 × 10^-7^ | 274 | *PAPLN* | Intron variant | US | Depression and suicide ideation | Laje et al. (2009) |
| Note: SAG= South Asian Genomes, SGDP=Simon Genome Diversity Project, SSIP=Stat Gen (Indians living in Singapore), 1000G SAS=1000 Genome South Asians,  WGP= Wellness Genome Project (Genomes of the Centenarians), PGA=Population Genetics of Andamanese, N.A=Not available (Ref: <http://clingen.igib.res.in/sage/>). | | | | | | | | | | | | |

**Table S3: Distribution of genotypes, allele frequencies and HW test of significance among cases and controls of the 16 selected SNPs in the present study.**

| **rs358592**  **(Risk=C)** | **Allele** | **Control** | **Case** | **rs1360550**  **(Risk=A)** | **Allele** | **Control** | **Case** |
| --- | --- | --- | --- | --- | --- | --- | --- |
|  | **TT** | 57(32.38%) | 84(64.12%) |  | **GG** | 56(35.89%) | 43(50.58%) |
|  | **CT** | 93(52.84%) | 40(30.53%) |  | **AG** | 91(58.33%) | 31(36.47%) |
|  | **CC** | 26(14.77%) | 7(5.34%) |  | **AA** | 9(5.76%) | 11(12.94%) |
|  | **N** | 176 | 131 |  | **N** | 156 | 85 |
|  | **T** | 0.588 | 0.794 |  | **G** | 0.651 | 0.688 |
|  | **C** | 0.412 | 0.206 |  | **A** | 0.349 | 0.312 |
|  | **HWX^2^** | 1.4464 | 0.5872 |  | **HWX^2^** | 12.5060*** | 1.9159 |
|  | **Heterozygosity**  **(Obs-Exp)** | 93-85= -8% | 40-43= -3% |  | **Heterozygosity**  **(Obs-Exp)** | 91-71= 20% | 31-37= -6% |
| **rs1109089**  **(Risk=T)** | **CC** | 103(57.22%) | 40(29.62%) | **rs1037448**  **(Risk=T)** | **CC** | 27(93.10%) | 36(70.58%) |
|  | **TC** | 55(30.55%) | 66(48.88%) |  | **TC** | 0 | 2(3.92%) |
|  | **TT** | 22(12.22%) | 29(21.48%) |  | **TT** | 2(6.90%) | 13(25.50%) |
|  | **N** | 180 | 135 |  | **N** | 29 | 51 |
|  | **C** | 0.725 | 0.5407 |  | **C** | 0.931 | 0.725 |
|  | **T** | 0.275 | 0.4593 |  | **T** | 0.069 | 0.275 |
|  | **HWX^2^** | 9.8322*** | 0.0332 |  | **HWX^2^** | 29*** | 41.4518*** |
|  | **Heterozygosity**  **(Obs-Exp)** | 55-72= -17% | 66-66= 0% |  | **Heterozygosity**  **(Obs-Exp)** | 0-4= -4% | 2-20= -18% |
| **rs4918918**  **(Risk=T)** | **CC** | 40(24.69%) | 35(34.31%) | **rs2576377**  **(Risk=C)** | **TT** | 159(90.34%) | 122(93.13%) |
|  | **TC** | 58(35.80%) | 40(39.21%) |  | **CT** | 17(9.66%) | 8(6.11%) |
|  | **TT** | 64(39.50%) | 27(26.47%) |  | **CC** | 0 NA | 1(0.76%) |
|  | **N** | 162 | 102 |  | **N** | 176 | 131 |
|  | **C** | 0.426 | 0.539 |  | **T** | 0.952 | 0.962 |
|  | **T** | 0.574 | 0.461 |  | **C** | 0.048 | 0.038 |
|  | **HWX^2^** | 11.6252*** | 4.5339* |  | **HWX^2^** | 0.4532 | 3.7085* |
|  | **Heterozygosity**  **(Obs-Exp)** | 58-79= -21% | 40-51= -11% |  | **Heterozygosity**  **(Obs-Exp)** | 17-16= 1% | 8-10= -2% |
| **rs10448044**  **(Risk=C)** | **TT** | 80(45.45%) | 38(30.15%) | **rs4751955**  **(Risk=A)** | **GG** | 90(61.64%) | 66(53.65%) |
|  | **CT** | 44(25.00%) | 20(15.87%) |  | **AG** | 41(28.08%) | 38(30.89%) |
|  | **CC** | 52(29.55%) | 68(53.96%) |  | **AA** | 15(10.27%) | 19(15.44%) |
|  | **N** | 176 | 126 |  | **N** | 146 | 123 |
|  | **T** | 0.580 | 0.381 |  | **G** | 0.757 | 0.691 |
|  | **C** | 0.420 | 0.619 |  | **A** | 0.243 | 0.309 |
|  | **HWX^2^** | 41.7445*** | 55.4628*** |  | **HWX^2^** | 8.2017** | 9.4016** |
|  | **Heterozygosity**  **(Obs-Exp)** | 44-86=-42% | 20-59=-39% |  | **Heterozygosity**  **(Obs-Exp)** | 41-54= -13% | 38-53= -15% |
| **rs4732812**  **(Risk=T)** | **CC** | 75(60.97%) | 16(19.27%) | **rs11143230**  **(Risk=C)** | **AA** | 77(75.49%) | 68(79.06%) |
|  | **TC** | 34(27.64%) | 54(65.06%) |  | **CA** | 15(14.70%) | 12(13.95%) |
|  | **TT** | 14(11.38%) | 13(15.66%) |  | **CC** | 10(9.80%) | 6(6.97%) |
|  | **N** | 123 | 83 |  | **N** | 102 | 86 |
|  | **C** | 0.748 | 0.518 |  | **A** | 0.828 | 0.860 |
|  | **T** | 0.252 | 0.482 |  | **C** | 0.172 | 0.140 |
|  | **HWX^2^** | 8.7574** | 7.6154** |  | **HWX^2^** | 23.7630*** | 15.0924*** |
|  | **Heterozygosity**  **(Obs-Exp)** | 34-46= -12% | 54-41= 13% |  | **Heterozygosity**  **(Obs-Exp)** | 15-29= -14% | 12-21= -9% |
| **rs10448042**  **(Risk=G)** | **AA** | 65(47.79%) | 31(43.05%) | **rs3851150**  **(Risk=C)** | **AA** | 148(88.09%) | 97(87.38%) |
|  | **GA** | 52(38.23%) | 10(13.88%) |  | **CA** | 19(11.30%) | 12(10.81%) |
|  | **GG** | 19(13.97%) | 31(43.05%) |  | **CC** | 1(0.59%) | 2(1.80%) |
|  | **N** | 136 | 72 |  | **N** | 168 | 111 |
|  | **A** | 0.771 | 0.500 |  | **A** | 0.937 | 0.9279 |
|  | **G** | 0.229 | 0.500 |  | **C** | 0.063 | 0.0721 |
|  | **HWX^2^** | 2.5342 | 37.5555*** |  | **HWX^2^** | 0.2046 | 4.0811* |
|  | **Heterozygosity**  **(Obs-Exp)** | 52-48= 4% | 10-36=-26% |  | **Heterozygosity**  **(Obs-Exp)** | 19-20= -1% | 12-15= -3% |
| **rs10997044**  **(Risk=A)** | **GG** | 103(91.96%) | 38(49.35%) | **rs10854398**  **(Risk=C)** | **TT** | 52(39.69%) | 25(31.25%) |
|  | **AG** | 6(5.35%) | 5(6.49%) |  | **CT** | 52(39.69%) | 38(47.5%) |
|  | **AA** | 3(2.67%) | 34(44.15%) |  | **CC** | 27(20.61%) | 17(21.25%) |
|  | **N** | 112 | 77 |  | **N** | 131 | 80 |
|  | **G** | 0.946 | 0.526 |  | **T** | 0.595 | 0.55 |
|  | **A** | 0.054 | 0.474 |  | **C** | 0.405 | 0.45 |
|  | **HWX^2^** | 24.9199*** | 58.2516*** |  | **HWX^2^** | 4.0624* | 0.1305 |
|  | **Heterozygosity**  **(Obs-Exp)** | 6-12=-6% | 5-39= -34% |  | **Heterozygosity**  **(Obs-Exp)** | 52-63= -11% | 38-40= -2% |
| **rs203136**  **(Risk=G)** | **TT** | 14(13.72%) | 25(37.31%) | **rs11628713**  **(Risk=T)** | **CC** | 58(39.28%) | 19(30.64%) |
|  | **GT** | 38(37.25%) | 11(16.41%) |  | **TC** | 72(48.64%) | 29(46.77%) |
|  | **GG** | 50(49.01%) | 31(46.26%) |  | **TT** | 18(12.16%) | 14(22.58%) |
|  | **N** | 102 | 67 |  | **N** | 148 | 62 |
|  | **T** | 0.324 | 0.455 |  | **C** | 0.635 | 0.540 |
|  | **G** | 0.676 | 0.545 |  | **T** | 0.365 | 0.460 |
|  | **HWX^2^** | 2.261 | 29.985*** |  | **HWX^2^** | 0.3647 | 0.2113 |
|  | **Heterozygosity**  **(Obs-Exp)** | 38-45= -7% | 11-33=-22% |  | **Heterozygosity**  **(Obs-Exp)** | 72-69= 3% | 29-31= -2% |

**Table S4: Network pathway percentage contribution of different default parameters of the figure S1.**

| **Pathway** | 26.19% |
| --- | --- |
|  |  |
| IMID | 25.62% |
| Pathway with 1,073 interactions from Pathway Commons |  |
|  |  |
| NCI_NATURE | 0.57% |
| Pathway with 10,122 interactions from Pathway Commons |  |
| **Co-expression** | 24.98% |
|  |  |
| Roth-Zlotnik-2006 | 7.07% |
| Gene expression analyses reveal molecular relationships among 20 regions of the human CNS. Roth et al (2006). *Neurogenetics* |  |
| Co-expression with 669,062 interactions from GEO |  |
|  |  |
| Innocenti-Brown-2011 | 4.79% |
| Identification, replication, and functional fine-mapping of expression quantitative trait loci in primary human liver tissue. |  |
| Innocenti et al (2011). *PLoS Genet* |  |
| Co-expression with 603,765 interactions from GEO |  |
|  |  |
| Burington-Shaughnessy-2008 | 2.68% |
| Tumor cell gene expression changes following short-term in vivo exposure to single agent chemotherapeutics are related to |  |
| survival in multiple myeloma. Burington et al (2008). *Clin Cancer Res* |  |
| Co-expression with 290,538 interactions from GEO |  |
|  |  |
| Wang-Maris-2006 | 1.86% |
| Integrative genomics identifies distinct molecular classes of neuroblastoma and shows that multiple genes are targeted by regional |  |
| alterations in DNA copy number. Wang et al (2006). *Cancer Res* |  |
| Co-expression with 264,023 interactions from GEO |  |
|  |  |
| Perou-Botstein-1999 | 1.59% |
| Distinctive gene expression patterns in human mammary epithelial cells and breast cancers. Perou et al (1999). *Proc Natl Acad* |  |
| *Sci U S A* |  |
| Co-expression with 65,069 interactions from supplementary material |  |
|  |  |
| Alizadeh-Staudt-2000 | 1.45% |
| Distinct types of diffuse large B-cell lymphoma identified by gene expression profiling. Alizadeh et al (2000). *Nature* |  |
| Co-expression with 90,336 interactions from supplementary material |  |
|  |  |
| Boldrick-Relman-2002 | 1.39% |
| Stereotyped and specific gene expression programs in human innate immune responses to bacteria. Boldrick et al (2002). *Proc* |  |
| *Natl Acad Sci U S A* |  |
| Co-expression with 111,707 interactions from supplementary material |  |
|  |  |
| Perou-Botstein-2000 | 1.18% |
| Molecular portraits of human breast tumours. Perou et al (2000). *Nature* |  |
| Co-expression with 185,068 interactions from supplementary material |  |
|  |  |
| Bild-Nevins-2006 B | 1.16% |
| Oncogenic pathway signatures in human cancers as a guide to targeted therapies. Bild et al (2006). *Nature* |  |
| Co-expression with 280,683 interactions from GEO |  |
|  |  |
| **Co-expression** | 24.98% |
|  |  |
| Wu-Garvey-2007 | 0.85% |
| The effect of insulin on expression of genes and biochemical pathways in human skeletal muscle. Wu et al (2007). *Endocrine* |  |
| Co-expression with 267,109 interactions from GEO |  |
|  |  |
| Dobbin-Giordano-2005 | 0.51% |
| Interlaboratory comparability study of cancer gene expression analysis using oligonucleotide microarrays. Dobbin et al (2005). |  |
| *Clin Cancer Res* |  |

Co-expression with 444,931 interactions from GEO

Wang-Cheung-2015

Genetic variation in insulin-induced kinase signaling. Wang et al (2015). *Mol Syst Biol* Co-expression with 411,047 interactions from GEO

**Shared protein domains**

INTERPRO

Shared protein domains with 608,863 interactions from InterPro

PFAM

Shared protein domains with 457,054 interactions from Pfam

**Physical Interactions**

IREF-DIP

Physical Interactions with 4,470 interactions from iRefIndex

BIOGRID-SMALL-SCALE-STUDIES

Physical Interactions with 58,871 interactions from BioGRID

IREF-PUBMED

Physical Interactions with 571 interactions from iRefIndex

IREF-BIND

Physical Interactions with 3,659 interactions from iRefIndex

**Predicted**

I2D-IntAct-Mouse2Human

The IntAct molecular interaction database in 2010. Aranda et al (2010). *Nucleic Acids Res* Predicted with 3,427 interactions from I2D

0.46%

20.48%

13.25%

7.23%

12.32%

5.83%

3.32%

2.89%

0.28%

9.75%

9.43%

| Wu-Stein-2010 | 0.32% |
| --- | --- |
| A human functional protein interaction network and its application to cancer data analysis. Wu et al (2010). *Genome Biol* |  |
| Predicted with 87,829 interactions from supplementary material |  |
| **Co-localization** | 6.28% |
|  |  |
| Schadt-Shoemaker-2004 | 4.22% |
| A comprehensive transcript index of the human genome generated using microarrays and computational approaches. Schadt et al |  |
| (2004). *Genome Biol* |  |
| Co-localization with 60,126 interactions from GEO |  |
|  |  |
| Johnson-Shoemaker-2003 | 2.06% |
| Genome-wide survey of human alternative pre-mRNA splicing with exon junction microarrays. Johnson et al (2003). *Science* |  |

| **Co-localization** | 6.28% |
| --- | --- |
|  |  |
| Johnson-Shoemaker-2003  Co-localization with 426,332 interactions from GEO |  |

**Figure S1: Pathway network analysis of 6 significant genes of the present study along with candidate genes of different known pathways of suicide behaviour. Default settings used for creation of network are as described in Table S4.**

**
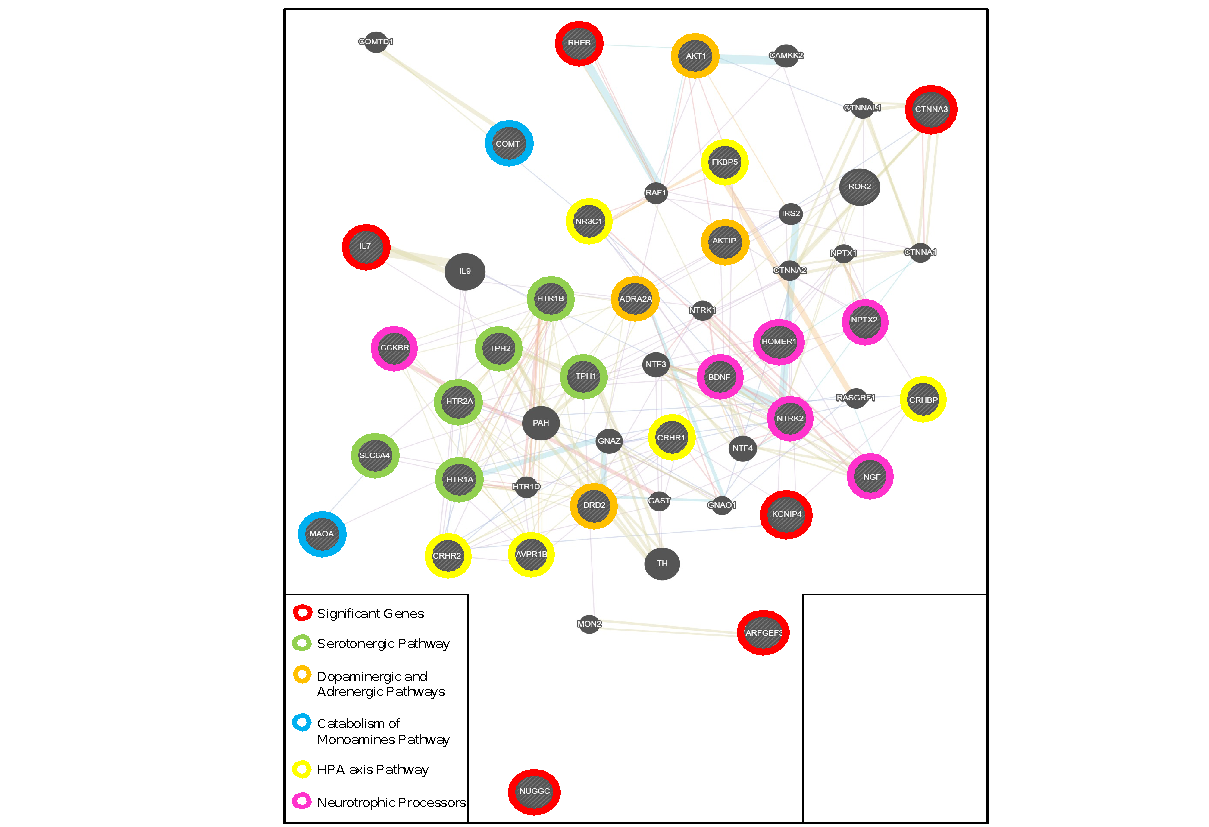
**
